# Evidence of initial success for China exiting COVID-19 social distancing policy after achieving containment

**DOI:** 10.12688/wellcomeopenres.15843.2

**Published:** 2020-10-01

**Authors:** Kylie E. C. Ainslie, Caroline E. Walters, Han Fu, Sangeeta Bhatia, Haowei Wang, Xiaoyue Xi, Marc Baguelin, Samir Bhatt, Adhiratha Boonyasiri, Olivia Boyd, Lorenzo Cattarino, Constanze Ciavarella, Zulma Cucunuba, Gina Cuomo-Dannenburg, Amy Dighe, Ilaria Dorigatti, Sabine L van Elsland, Rich FitzJohn, Katy Gaythorpe, Azra C Ghani, Will Green, Arran Hamlet, Wes Hinsley, Natsuko Imai, David Jorgensen, Edward Knock, Daniel Laydon, Gemma Nedjati-Gilani, Lucy C Okell, Igor Siveroni, Hayley A Thompson, H. Juliette T. Unwin, Robert Verity, Michaela Vollmer, Patrick G T Walker, Yuanrong Wang, Oliver J Watson, Charles Whittaker, Peter Winskill, Christl A Donnelly, Neil M Ferguson, Steven Riley

**Affiliations:** 1 MRC Centre for Global Infectious Disease Analysis, Imperial College London, London, W2 1PG, UK; 2 Department of Mathematics, Imperial College London, London, SW7 2AZ, UK; 3 NIHR Health Protection Research Unit in Healthcare Associated Infections and Antimicrobial Resistance, Imperial College London, London, SW7 2AZ, UK; 4 Department of Laboratory Medicine and Pathology, Brown University, Providence, RI, 02912, USA; 5 Department of Statistics, University of Oxford, Oxford, OX1 3LB, UK

**Keywords:** COVID-19, social distancing, exit strategy, transmission

## Abstract

**Background**: The COVID-19 epidemic was declared a Global Pandemic by WHO on 11 March 2020. By 24 March 2020, over 440,000 cases and almost 20,000 deaths had been reported worldwide. In response to the fast-growing epidemic, which began in the Chinese city of Wuhan, Hubei, China imposed strict social distancing in Wuhan on 23 January 2020 followed closely by similar measures in other provinces. These interventions have impacted economic productivity in China, and the ability of the Chinese economy to resume without restarting the epidemic was not clear.

**Methods**: Using daily reported cases from mainland China and Hong Kong SAR, we estimated transmissibility over time and compared it to daily within-city movement, as a proxy for economic activity.

**Results**: Initially, within-city movement and transmission were very strongly correlated in the five mainland provinces most affected by the epidemic and Beijing. However, that correlation decreased rapidly after the initial sharp fall in transmissibility. In general, towards the end of the study period, the correlation was no longer apparent, despite substantial increases in within-city movement. A similar analysis for Hong Kong shows that intermediate levels of local activity were maintained while avoiding a large outbreak. At the very end of the study period, when China began to experience the re-introduction of a small number of cases from Europe and the United States, there is an apparent up-tick in transmission.

**Conclusions:** Although these results do not preclude future substantial increases in incidence, they suggest that after very intense social distancing (which resulted in containment), China successfully exited its lockdown to some degree. Elsewhere, movement data are being used as proxies for economic activity to assess the impact of interventions. The results presented here illustrate how the eventual decorrelation between transmission and movement is likely a key feature of successful COVID-19 exit strategies.

## Introduction

The COVID-19 epidemic was declared a Global Pandemic by the World Health Organization on 11 March 2020 ^1^. By 24 March 2020, over 440,000 cases and almost 20,000 deaths had been reported worldwide. The outbreak began in the Chinese city of Wuhan, Hubei in December 2019. In response to the fast-growing epidemic, the Chinese government implemented strict social distancing measures to halt the spread of COVID-19, with a city-wide lockdown (including closing non-essential businesses and public transport, and restricting individual movement) first implemented in Wuhan, Hubei on 23 January 2020 ^2, 3^. Similar social distancing measures were enacted soon after in other provinces.

With the exception of Hubei Province, companies and factories began reopening on 10 February ^4^. On 11 March, businesses began reopening in Hubei ^5^ and, on 12 March, Hubei provincial government announced a series of measures to gradually resume transportation ^6, 7^. For the first time since the outbreak began there have been no new confirmed cases (with no known contact with an imported case) caused by local transmission in mainland China reported for five consecutive days up to 23 March 2020 ^8– 11^. At the peak of the outbreak in China (early February), there were between 2,000 and 4,000 new confirmed cases per day. The lack of new confirmed cases caused by local transmission is an indication that the social distancing measures enacted in China have led to control of COVID-19.

Social distancing measures have impacted economic productivity in China and it is currently unclear whether the Chinese economy can resume without restarting the epidemic. Similar to mainland China, the Hong Kong government implemented border restrictions, remote working arrangements, and school closures ^11^, but did not stop economic activity to the same degree.

**Figure 1.**
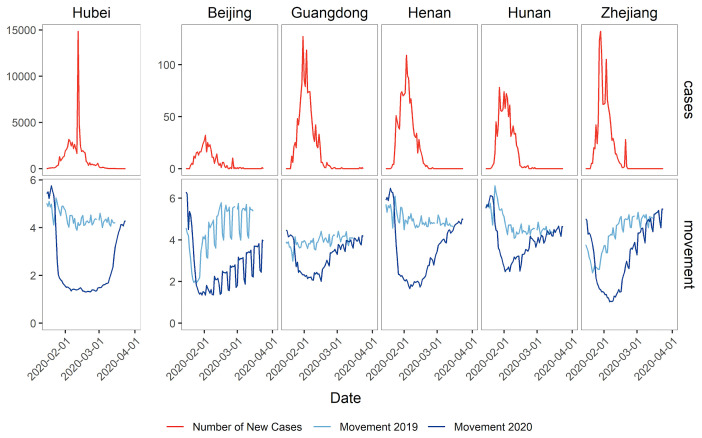
Plots of daily new confirmed cases (red line, top row) and daily movement index (Exante Data Inc, NY, bottom row) for Hubei, Beijing, Guangdong, Henan, Hunan, and Zhejiang in 2019 (light blue) and 2020 (dark blue). Daily new confirmed cases are shown by report date. Movement data in 2019 have been adjusted so that the first day of Lunar New Year in 2019 is assumed to be on the same Gregorian date as 2020. The cyclic movement patterns seen in Beijing and toward the end of February in Zhejiang are the result of decreased travel on weekends.

Here, we use daily reported COVID-19 cases for each province in mainland China and for Hong Kong SAR ^11^ ( Figure 1) and within-city movement data to examine the temporal correlation of transmission and economic activity.

## Methods

The reproduction number ( *R _t_*) measures transmissibility and is defined as the average number of new cases generated by each case. When the number of cases is growing, *R _t_* is greater than 1; when the number of cases is decreasing, *R _t_* is less than 1. Changes in *R _t_* are not immediately evident in case data for two reasons. First, there are delays from infection to the onset of symptoms and from the onset of symptoms to seeking care. Second, people must be tested, and those with positive test results must be reported to become a case in these data. We compare estimates of *R _t_* with daily within-city movement data, used as a proxy for economic activity, to evaluate the relationship between economic activity and control of COVID-19.

We obtained daily confirmed cases over 16 January to 24 March 2020 from the dashboard maintained by Chinese Center for Disease Prevention and Control (CCDC) ^11^. The CCDC dashboard collates numbers of confirmed cases reported by national and local health commissions in each province in mainland China, and Hong Kong SAR and Macau SAR. Confirmed cases are defined as suspected cases, who have epidemiological links and/or clinical symptoms, and are detected with SARS-CoV-2 by PCR tests. However, in Hubei province, clinically diagnosed cases were additionally included between 12 and 19 February ^12^. Imported cases were excluded.

We obtained daily within-city movement data, used as a proxy for economic activity, from 1 January to 24 March 2020 for major metropolitan cities within each province in mainland China ( Figure 1), Hong Kong SAR, and Macau SAR. These data, provided by Exante Data Inc ^13^, measured travel activity relative to the 2019 average (excluding Lunar New Year). The underlying data are based on near real-time people movement statistics from Baidu. Based on GPS tracking, the data allow quantification of the number of trips taken per person in the population. At the country level, approximately five trips per person per day was normal. If that went down to three trips per person per day, that would be described as a 40% drop. We calculated the weighted average movement within each province using city population size (Table S1, *Extended data* ^14^). 

Estimates of *R _t_* over time for each region were obtained using the EpiEstim R package ^15^. Briefly, at each time step t, R _t_ is the average reproduction number over the time window *t – τ*, where *τ = 7* days. We use confirmed daily case counts to estimate R _t_, thus we estimate the average number of new cases generated by cases with symptom onset at time *t*. We assumed a mean serial interval of 6.48 days with a standard deviation of 3.83 days ^16^. Subsequent investigation of the serial interval of COVID-19 has confirmed that 6.5 days is a reasonable mean serial interval ^17^. To account for the delay between symptom onset and report of confirmed cases, we calculated the cross-correlation between daily movement and *R _t_* for Hubei province during the peak of the epidemic (before 15 February 2020) for time lags between 0 and 10 days. During the peak of the epidemic, Hubei Province had 82% of all confirmed cases in mainland China, Hong Kong SAR, and Macau SAR. Cross-correlations were calculated using the ccf function in the stats R package. The highest correlation was observed for a 4-day lag (Figure S1, *Extended data* ^14^). *R _t_* dates were backdated according to the assumed lag. The implementation of a lag is designed to account for reporting delay of cases rather than the time between symptom onset in a case and the subsequent onset of symptoms in someone they have infected. Next, we determined biweekly rolling Pearson correlation coefficients between *R _t_* and movement data for each province.

 To determine how the movement patterns in Hubei province (where the most cases were observed) influenced the *R _t_* in other regions, we calculated biweekly rolling Pearson correlation coefficients between *R _t_* in each region and movement in Hubei. All analyses were performed in R 3.6.2 ^18^. The open source package pika is available from GitHub https://github.com/mrc-ide/pika, with a detailed accompanying vignette covering the main implementation presented here.

## Results

Both daily cases and within-city movement exhibited similar patterns in the five most affected provinces and in Beijing ( Figure 1). Hubei had the largest number of reported cases, and the largest, longest-lasting reduction in within-city movement. Beijing and the other four provinces had much smaller epidemics and restarted within-city movements after two weeks to some degree. A weekday effect was especially evident in Beijing with substantially lower levels of movement at the weekend. Mean within-city movement in Hunan never dropped below two journeys per day.

**Figure 2.**
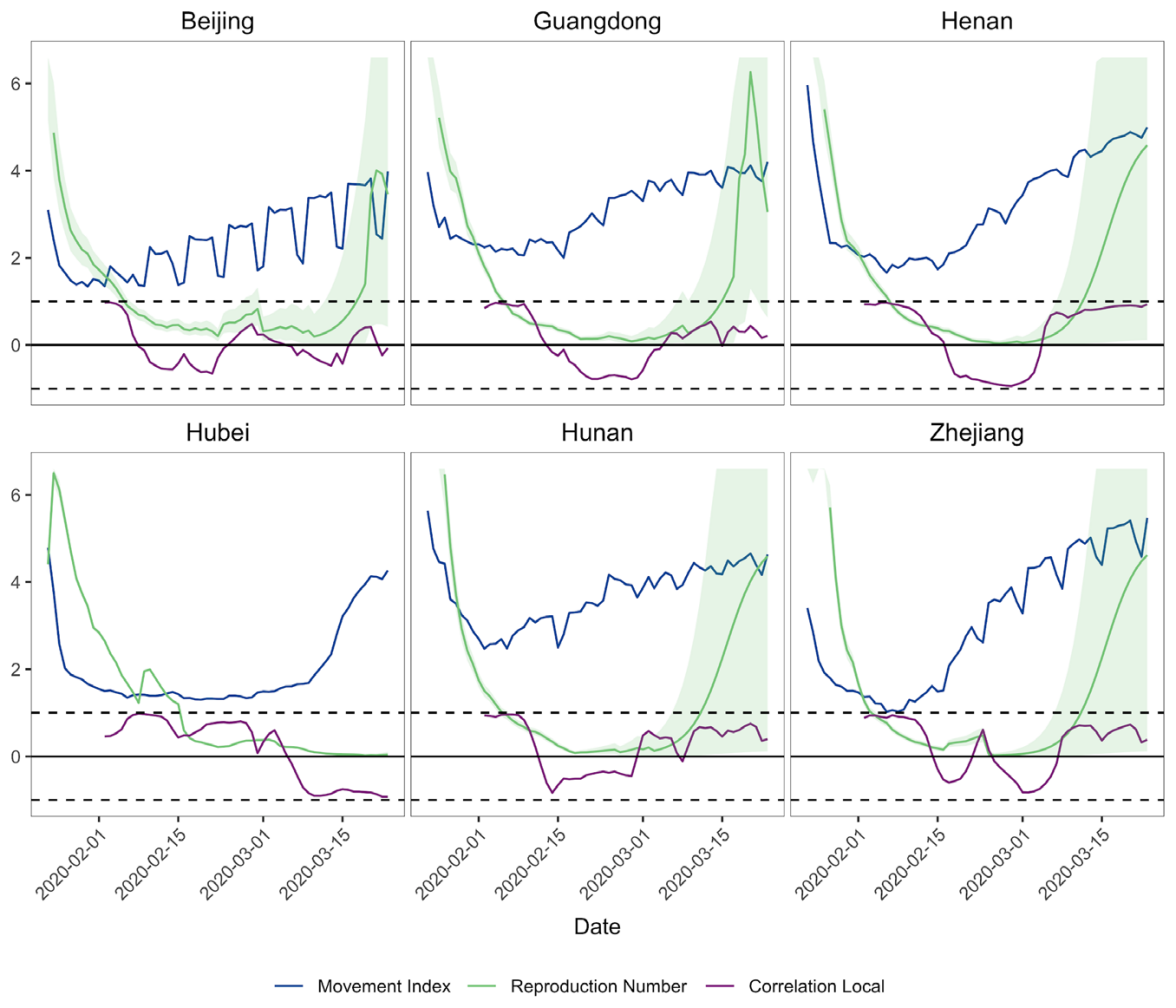
Plots of estimated reproduction number, movement, and correlation in the five provinces in mainland China with the highest numbers of cumulative confirmed cases and Beijing (top: Beijing, Guangdong, Henan; bottom: Hubei, Hunan, Zhejiang). Blue: mean daily movement index (Exante Data Inc, NY), green: mean effective reproduction number estimated using daily confirmed case reports (green shading: 95% credible interval), purple: local correlation between movement index and effective reproduction number. Reproduction number was estimated assuming a lag of -4 days. Dashed lines indicate the upper and lower bounds of the correlation coefficients (-1, 1).

As movement restrictions were put into place within mainland China from late January to early February 2020, within-city movement and *R _t_* were highly positively correlated ( Figure 2). That is, a decrease in movement was highly correlated with a decrease in *R _t_*. However, as movement resumed within each province/region, the correlation between within-city movement and *R _t_* declined steeply and became negative for a substantial period. At the end of the period, there was a slight increase in *R _t_* driven by a small number of cases. Although these were most likely cases with direct contact with imported cases, based on press reports, we were not able to differentiate cases caused by local transmission from those caused by imported cases in these data. Therefore, these final up-ticks in *R _t_* are an upper bound on transmission.

Although it is possible that the epidemic in Wuhan drove patterns elsewhere, if this were the case it also rapidly diminished once transmissibility dropped. We evaluated the correlation between within-city movement in Hubei and *R _t_* in other regions (Figure S2, *Extended data* ^14^). Movement in Hubei was initially strongly positively correlated with *R _t_* in other provinces/regions. However, as movement resumed within each province/region, these correlations between within-city movement in Hubei and *R _t_* elsewhere became weaker.

**Figure 3.**
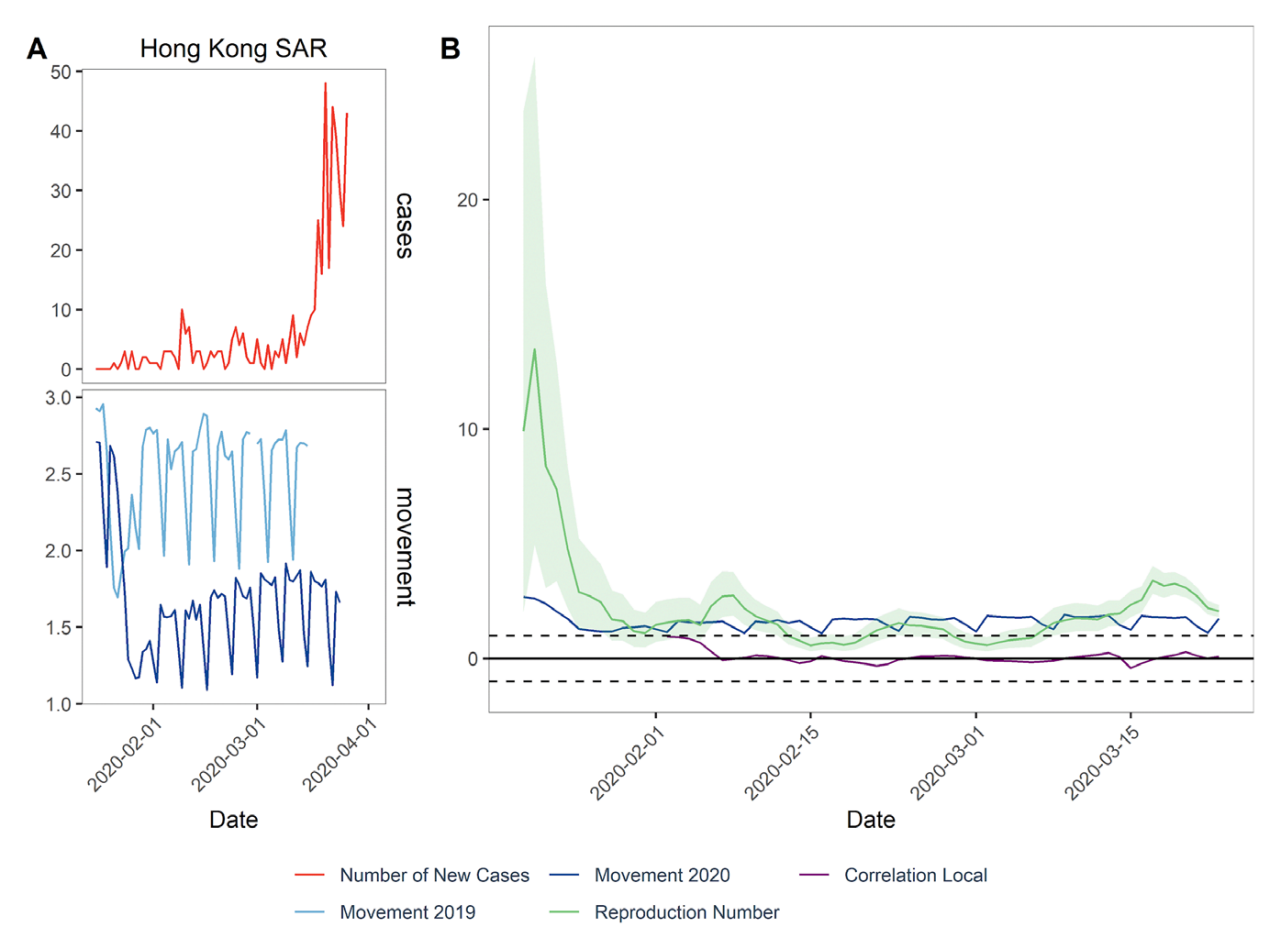
( **A**) Plots of daily new confirmed cases (top, red line) and daily movement index (bottom) for Hong Kong in 2019 (light blue) and 2020 (dark blue). Daily new confirmed cases are shown by report date. Movement data in 2019 have been adjusted so that the first day of Lunar New Year in 2019 is assumed to be on the same Gregorian date as 2020. The cyclic movement patterns are the result of decreased travel on weekends. ( **B**) Plots of estimated reproduction number, movement, and correlation in Hong Kong. Dark blue: mean daily movement index, green: mean effective reproduction number estimated using daily confirmed case reports (green shading: 95% credible interval), purple: local correlation between movement index and effective reproduction number. Dashed lines indicate the upper and lower bounds of the correlation coefficients (-1, 1).

In Hong Kong SAR, where less strict movement restrictions were implemented and a lessened, but consistent level of economic activity has been maintained, we observed no correlation between intra-Hong Kong movement and *R _t_* ( Figure 3). We observed a high R *_t_* value in January with very wide confidence intervals. This is due to a lack of data prior to January. We recognise this is a limitation of our approach and the R package EpiEstim used to estimate R *_t_*.

As a sensitivity analysis, we calculated region-specific optimal lags to see if using a different lag in each region impacted the estimated correlation between *R _t_* and movement. Optimal lags were similar. For three regions, the optimal lag was 0 days, for one region it was -1 and for two regions it was -4 days.

## Discussion

We assessed the correlation between daily movement and estimated *R _t_* over time. We observed strong positive correlation between movement and *R _t_* initially followed by a drop in this correlation as China began to remove movement restrictions and restart their economy. These results provide evidence that China’s containment strategies are continuing to be effective as they restart their economy. ****

This work is an analysis of correlation, not causation. While within-city movement undoubtedly affects *R _t_*, this analysis does not infer causation. To estimate *R _t_*, we used confirmed case reports; however, confirmed cases are only a proportion of the total number of infected individuals. Therefore, our estimates of *R _t_* may be biased if the proportion of cases being detected varied substantially over short periods of time.

These results should be considered when other countries use movement data to assess the impact of disease control interventions. While reductions in movement appear to be necessary in the short term, it appears that China rapidly managed to restart key elements of economic activity without increasing transmission. Therefore, while movement data are important, the decorrelation between movement and transmission becomes a goal for any exit strategy.

## Data availability

### Underlying data

Zenodo: mrc-ide/china-exit-covid-19: Second release. https://doi.org/10.5281/zenodo.3751005 ^14^

This project contains the following underlying data:

- archive/china_extract_new_case_data/china_new_case_data.csv (daily confirmed cases in China by province from the CCDC dashboard ^11^)
- archive/china_read_exante_data/exante_movement_data.csv (daily within-city movement data from Exante ^13^)

### Extended data

Zenodo: mrc-ide/china-exit-covid-19: Second release. https://doi.org/10.5281/zenodo.3751005 ^14^

This project contains the following extended data:

- china_exit_supp_mat.pdf (supplementary material containing Table S1, Figure S1 and Figure S2)

Data are available under the terms of the Creative Commons Zero "No rights reserved" data waiver (CC0 1.0 Public domain dedication).

### Code availability

Reproducible code is available at: https://github.com/mrc-ide/china-exit-covid-19

Archived code at time of publication: https://doi.org/10.5281/zenodo.3751005 ^14^

License: Creative Commons Zero "No rights reserved" data waiver (CC0 1.0 Public domain dedication)
