# Supplementary material for: Evidence of initial success for China exiting COVID-19 social distancing policy after achieving containment
Source: Wellcome Open Res. 2020 Oct 1;5:81. Originally published 2020 Apr 28. [Version 2] doi: 10.12688/wellcomeopenres.15843.2 (PMC7236587; doi:10.12688/wellcomeopenres.15843.2)
Supplement: Peer review history

# Peer review: Reviewer response for version 2

**DOI:** 10.21956/wellcomeopenres.17946.r40676

**Published:** 2020-10-02

**Authors:** Alex R. Cook

**Affiliations:** 1 Saw Swee Hock School of Public Health, National University of Singapore, Singapore, Singapore

No further comments, thank you.

Is the work clearly and accurately presented and does it cite the current literature?

Yes

If applicable, is the statistical analysis and its interpretation appropriate?

Yes

Are all the source data underlying the results available to ensure full reproducibility?

Yes

Is the study design appropriate and is the work technically sound?

Yes

Are the conclusions drawn adequately supported by the results?

Yes

Are sufficient details of methods and analysis provided to allow replication by others?

Yes

Reviewer Expertise:

Modelling.

I confirm that I have read this submission and believe that I have an appropriate level of expertise to confirm that it is of an acceptable scientific standard.

# Peer review: Reviewer response for version 1

**DOI:** 10.21956/wellcomeopenres.17379.r38549

**Published:** 2020-05-18

**Authors:** Sheila Bird

**Affiliations:** 1 MRC Biostatistics Unit, School of Clinical Medicine, University of Cambridge, Cambridge, UK

Transmissibility over time (mainland China and Hong Kong) is estimated and compared to daily within-city movement. The two were initially strong correlated but correlation reduced rapidly in lock-down after the initial sharp fall in transmissibility. Within-city movement then picked up but remained de-correlated from transmission, at least initially until China experienced re-introductions of COVID cases. Hong Kong maintained intermediate levels of local activity without a large outbreak (but beware infections having run wild in camps of migrant workers in Singapore and similar concerns in Qatar).

 Key conclusion that could have international resonance is that maintenance of de-correlation between transmission and movement is a goal for countries to monitor and achieve as they emerge from lock-down.

 City-wide strict social distancing in Wuhan, Hubei was implemented on 23 Jan 2020; and soon after in other provinces where re-opening began on 10 February (Hubei excepted until 11/12 March). For 5 consecutive days up to 23 March there were no cases arising from local (vs imported) transmission.

 Paper refers to "daily reported cases" for each mainland province in China and for Hong Kong. Report-date is generally later than onset-date or swab-date, as acknowledged in the paper. Greater clarity would be helpful about which case-date is displaying in Fig 1. I assume report-date. For case definition, see paragraph 1 on page 5. Movement data for major metropolitan cities are described in paragraph 2: travel activity (from Exante Data Inc) measured relative to 2019-average [excluding Lunar New Year]. Underlying data based on "near real-time people movement statistics from BAIDU". Are such data available internationally? **Five trips per person per day was normal.**

 Dramatically, Fig 1 displays the difference in movement data, day by day, between 2019 and 2020 as well as the report-date profile of new cases, from which, using EpiEstim R package, reproduction number over time was estimated by assuming mean serial interval of 6.5 days (sd 3.8 days). To account for delay between symptom-onset-date and confirmed-case-report-date, lagged correlations (0 to 10 days of lag) with movement data in Hubei were investigated up to case-peak in 15 Feb. 2020. **Highest correlation: 4-day lag **but with disconcerting variation across provinces.

 Weekday effect on movement was apparent in Beijing with **substantially lower levels of movement at weekends**. Weekend effects on deaths in UK may not be solely due to reporting artefacts... Hong Kong's different approach (movements allowed to increase back gradually) is illustrated in Fig 3.

 Authors summarize: de-correlation between movement and transmission becomes a goal for any exit strategy. 

 This paper - about learning from international data - is hugely important, succinct and well-written.

Is the work clearly and accurately presented and does it cite the current literature?

Yes

If applicable, is the statistical analysis and its interpretation appropriate?

Yes

Are all the source data underlying the results available to ensure full reproducibility?

Yes

Is the study design appropriate and is the work technically sound?

Yes

Are the conclusions drawn adequately supported by the results?

Yes

Are sufficient details of methods and analysis provided to allow replication by others?

Yes

Reviewer Expertise:

Biostatistics including transmissible infectious disease epidemiology.

I confirm that I have read this submission and believe that I have an appropriate level of expertise to confirm that it is of an acceptable scientific standard.

# Response

**Published:** 2020-09-16

**Authors:** Kylie Ainslie

*We would like to thank both reviewers for their comments. We found them helpful for improving the manuscript. Below we respond to each of the reviewers comments individually. We apologise for the delay in addressing these comments. Responses to reviewer comments are below in italics.*

Transmissibility over time (mainland China and Hong Kong) is estimated and compared to daily within-city movement. The two were initially strong correlated but correlation reduced rapidly in lock-down after the initial sharp fall in transmissibility. Within-city movement then picked up but remained de-correlated from transmission, at least initially until China experienced re-introductions of COVID cases. Hong Kong maintained intermediate levels of local activity without a large outbreak (but beware infections having run wild in camps of migrant workers in Singapore and similar concerns in Qatar).

Key conclusion that could have international resonance is that maintenance of de-correlation between transmission and movement is a goal for countries to monitor and achieve as they emerge from lock-down.

City-wide strict social distancing in Wuhan, Hubei was implemented on 23 Jan 2020; and soon after in other provinces where re-opening began on 10 February (Hubei excepted until 11/12 March). For 5 consecutive days up to 23 March there were no cases arising from local (vs imported) transmission.

Paper refers to "daily reported cases" for each mainland province in China and for Hong Kong. Report-date is generally later than onset-date or swab-date, as acknowledged in the paper. Greater clarity would be helpful about which case-date is displaying in Fig 1. I assume report-date. For case definition, see paragraph 1 on page 5. Movement data for major metropolitan cities are described in paragraph 2: travel activity (from Exante Data Inc) measured relative to 2019-average [excluding Lunar New Year]. Underlying data based on "near real-time people movement statistics from BAIDU". Are such data available internationally? Five trips per person per day was normal.

*Figure 1 is displaying daily case counts by report date. We have updated the figure 1 legend to make this more clear.* *Movement data is available for many countries from Facebook Data for Good. More information can be found here https://dataforgood.fb.com/*

Dramatically, Fig 1 displays the difference in movement data, day by day, between 2019 and 2020 as well as the report-date profile of new cases, from which, using EpiEstim R package, reproduction number over time was estimated by assuming mean serial interval of 6.5 days (sd 3.8 days). To account for delay between symptom-onset-date and confirmed-case-report-date, lagged correlations (0 to 10 days of lag) with movement data in Hubei were investigated up to case-peak in 15 Feb. 2020. Highest correlation: 4-day lag but with disconcerting variation across provinces.

Weekday effect on movement was apparent in Beijing with substantially lower levels of movement at weekends. Weekend effects on deaths in UK may not be solely due to reporting artefacts... Hong Kong's different approach (movements allowed to increase back gradually) is illustrated in Fig 3.

Authors summarize: de-correlation between movement and transmission becomes a goal for any exit strategy. 

This paper - about learning from international data - is hugely important, succinct and well-written.

# Peer review: Reviewer response for version 1

**DOI:** 10.21956/wellcomeopenres.17379.r38550

**Published:** 2020-05-18

**Authors:** Alex R. Cook

**Affiliations:** 1 Saw Swee Hock School of Public Health, National University of Singapore, Singapore, Singapore

This is a nice, short article by Ainslie *et al*. I have only a few comments the authors might wish to address:

- It might be worth updating the case counts and deaths in the introduction.
- Please clarify your definition of Rt in the methods. It is the average number of new cases generated by all cases INFECTED at time t, or with ONSET on time t, or it is the average number of new cases INFECTED at time t from active cases at time t, or it is the average number of new ONSETS on time t, from active cases some time before that?
- Your serial interval seems a little high. Does the lag for the correlations support an interval with mean/median 4d instead?
- Rt for HK in January seems very high. Please double check the data for this.
- I found Figure 2 really difficult to parse, with three quite incongruent data types being presented. Perhaps if the correlations were slightly offset it would be easier to visualize? I have similar difficulties with F3b.

Thank you for giving me the opportunity to read this interesting paper.

Is the work clearly and accurately presented and does it cite the current literature?

Yes

If applicable, is the statistical analysis and its interpretation appropriate?

Yes

Are all the source data underlying the results available to ensure full reproducibility?

Yes

Is the study design appropriate and is the work technically sound?

Yes

Are the conclusions drawn adequately supported by the results?

Yes

Are sufficient details of methods and analysis provided to allow replication by others?

Yes

Reviewer Expertise:

Modelling.

I confirm that I have read this submission and believe that I have an appropriate level of expertise to confirm that it is of an acceptable scientific standard.

# Response

**Published:** 2020-09-16

**Authors:** Kylie Ainslie

We would like to thank both reviewers for their comments. We found them helpful for improving the manuscript. Below we respond to each of the reviewers comments individually. We apologise for the delay in addressing these comments. Below are our responses to reviewer comments in italics.

This is a nice, short article by Ainslie et al. I have only a few comments the authors might wish to address:

It might be worth updating the case counts and deaths in the introduction.

*We only have movement data up to 24 March, 2020, thus we are unable to extend the analysis to dates beyond March 24. We feel it would cause unnecessary confusion to discuss the number of cases and deaths beyond the end date of this analysis.*

Please clarify your definition of Rt in the methods. It is the average number of new cases generated by all cases INFECTED at time t, or with ONSET on time t, or it is the average number of new cases INFECTED at time t from active cases at time t, or it is the average number of new ONSETS on time t, from active cases some time before that?

*To clarify the methods, we have added the following text to the 4th paragraph of the Methods section:*

*Estimates of Rt over time for each region were obtained using the EpiEstim R package [1] . Briefly, at each time step t, Rt is the average reproduction number over the time window t-τ, where τ=7 days. We use confirmed daily case counts to estimate Rt, thus we estimate the average number of new cases generated by cases with symptom onset at time t.*

Your serial interval seems a little high. Does the lag for the correlations support an interval with mean/median 4d instead?

*Subsequent investigation of the serial interval of COVID-19 has confirmed that 6.5 days is a reasonable mean serial interval [2]. Additionally, our lag is designed to account for reporting delay of cases rather than the time between symptom onset in a case and the subsequent onset of symptoms in someone they have infected. We have altered the text (below) to make this clearer:*

*We assumed a mean serial interval of 6.48 days with a standard deviation of 3.83 days [3]. Subsequent investigation of the serial interval of COVID-19 has confirmed that 6.5 days is a reasonable mean serial interval [2].To account for the delay between symptom onset and report of confirmed cases, we calculated the cross-correlation between daily movement and Rt for Hubei province during the peak of the epidemic (before 15 February 2020) for time lags between 0 and 10 days. During the peak of the epidemic, Hubei Province had 82% of all confirmed cases in mainland China, Hong Kong SAR, and Macau SAR. Cross-correlations were calculated using the ccf function in the stats R package. The highest correlation was observed for a 4-day lag (Figure S1, Extended data [4] ). Rt dates were backdated according to the assumed lag. The implementation of a lag is designed to account for reporting delay of cases rather than the time between symptom onset in a case and the subsequent onset of symptoms in someone they have infected.* 

Rt for HK in January seems very high. Please double check the data for this.

*The Rt value for January is indeed high, but also has very wide confidence intervals. This is due to a lack of data prior January. We recognise that this is a limitation of our approach and EpiEstim, the R package that we used to estimate Rt. We have acknowledged this in our results section with the following text:*

*We observed a high Rt value in January with very wide confidence intervals. This is due to a lack of data prior to January. We recognise this is a limitation of our approach and the R package EpiEstim used to estimate Rt.*

I found Figure 2 really difficult to parse, with three quite incongruent data types being presented. Perhaps if the correlations were slightly offset it would be easier to visualize? I have similar difficulties with F3b.

*We attempted different figure configurations, but none were satisfactory, so we’ve left the original figures.*
